# Ampullary Neuroendocrine Neoplasms: Identification of Prognostic Factors in a Multicentric Series of 119 Cases

**DOI:** 10.1007/s12022-022-09720-6

**Published:** 2022-05-13

**Authors:** Alessandro Vanoli, Oneda Grami, Catherine Klersy, Anna Caterina Milanetto, Luca Albarello, Matteo Fassan, Claudio Luchini, Federica Grillo, Paola Spaggiari, Frediano Inzani, Silvia Uccella, Paola Parente, Gennaro Nappo, Paola Mattiolo, Massimo Milione, Andrea Pietrabissa, Lorenzo Cobianchi, Marco Schiavo Lena, Stefano Partelli, Antonio Di Sabatino, Christine Sempoux, Carlo Capella, Claudio Pasquali, Claudio Doglioni, Fausto Sessa, Aldo Scarpa, Guido Rindi, Marco Paulli, Alessandro Zerbi, Massimo Falconi, Enrico Solcia, Stefano La Rosa

**Affiliations:** 1 Anatomic Pathology Unit, Department of Molecular Medicine, University of Pavia, Via Carlo Forlanini 16 – 27100, Pavia, Italy; 2 Anatomic Pathology, Fondazione IRCCS San Matteo Hospital, Pavia, Italy; 3 Biometry and Clinical Epidemiology Service, Fondazione IRCCS Policlinico San Matteo, Pavia, Italy; 4 Department of Surgery, Oncology and Gastroenterology-DiSCOG, University of Padova, Padua, Italy; 5 Pathology Unit, IRCCS San Raffaele Scientific Institute, Milan, Italy; 6 Department of Medicine (DIMED), Surgical Pathology Unit, University of Padua, Padua, Italy; 7 Veneto Institute of Oncology, IOV-IRCCS, Padua, Italy; 8 Department of Diagnostics and Public Health, Section of Pathology, and ARC-Net Research Centre, University of Verona, Verona, Italy; 9 Department of Surgical and Diagnostic Sciences, Pathology Unit, University of Genoa, Genoa, Italy; 10 Pathology Department, Humanitas Clinical and Research Center-IRCCS Rozzano, Milan, Italy; 11 Department of Woman and Child Health Sciences and Public Health, Anatomic Pathology Unit, Fondazione Policlinico Universitario A. Gemelli – IRCCS, Rome, Italy; 12 Rome ENETS Center of Excellence, Rome, Italy; 13 Department of Medicine and Surgery, Unit of Pathology, University of Insubria, Varese, Italy; 14 Unit of Pathology, Fondazione IRCCS Ospedale Casa Sollievo Della Sofferenza, San Giovanni Rotondo (FG), Italy; 15 Department of Biomedical Sciences, Humanitas University, Pieve Emanuele, Italy; 16 IRCCS Humanitas Research Hospital, Rozzano, Italy; 17 Department of Diagnostics and Public Health, Section of Pathology, University of Verona, Verona, Italy; 18 Diagnostic Pathology and Laboratory Medicine Department, Fondazione IRCCS Istituto Nazionale Dei Tumori, ENETS Center of Excellence, Milan, Italy; 19 General Surgery Unit, IRCCS Policlinico San Matteo Foundation, Pavia, Italy; 20 Pancreatic Surgery Unit, Pancreas Translational & Clinical Research Center, San Raffaele Scientific Institute IRCCS, Milan, Italy; 21 Department of Internal Medicine, IRCCS San Matteo Hospital Foundation, University of Pavia, Pavia, Italy; 22 Service of Clinical Pathology, Institute of Pathology, Lausanne University Hospital and University of Lausanne, Lausanne, Switzerland; 23 Department of Life Sciences, Section of Anatomic Pathology, Università Cattolica del Sacro Cuore, Rome, Italy

**Keywords:** Major ampulla, Minor papilla, Neuroendocrine tumor, Neuroendocrine carcinoma, Tumor grade

## Abstract

Neuroendocrine neoplasms (NENs) of the major and minor ampulla are rare diseases with clinico-pathologic features distinct from non-ampullary-duodenal NENs. However, they have been often combined and the knowledge on prognostic factors specific to ampullary NENs (Amp-NENs) is limited. The aim of this study was to identify factors associated with metastatic potential and patient prognosis in Amp-NENs. We clinically and histologically investigated an international series of 119 Amp-NENs, comprising 93 ampullary neuroendocrine tumors (Amp-NETs) and 26 neuroendocrine carcinomas (Amp-NECs). Somatostatin-producing tubulo-acinar NET represented the predominant Amp-NET histologic subtype (58 cases, 62%, 12 associated with type 1 neurofibromatosis). Compared to Amp-NETs, Amp-NECs arose in significantly older patients and showed a larger tumor size, a more frequent small vessel invasion, a deeper level of invasion and a higher rate of distant metastasis, and, importantly, a tremendously worse disease-specific patient survival. In Amp-NETs, the WHO grade proved to be a strong predictor of disease-specific survival (hazard ratio: 12.61, *p* < 0.001 for G2 vs G1), as well as patient age at diagnosis > 60 years, small vessel invasion, pancreatic invasion, and distant metastasis at diagnosis. Although nodal metastatic disease was not associated with survival by itself, patients with > 3 metastatic lymph nodes showed a worse outcome in comparison with the remaining Amp-NET cases with lymphadenectomy. Tumor epicenter in the major ampulla, small vessel invasion, and tumor size > 16 mm were independent predictors of nodal metastases in Amp-NETs. In conclusion, we identified prognostic factors, which may eventually help guide treatment decisions in Amp-NENs.

## Introduction

Duodenal neuroendocrine neoplasms (NENs) may be distinguished in ampullary and non-ampullary NENs, depending on their site. Ampullary neuroendocrine neoplasms (Amp-NENs) are rare malignancies, accounting for about 20% of all duodenal NENs [1]. These neoplasms show clinical, histologic, and immunohistochemical distinctive features in comparison with non-ampullary duodenal NENs (non-Amp-Duo-NENs) [1, 2]. Indeed, Amp-NENs are larger and more commonly present with abdominal pain or jaundice due to bile duct obstruction [3, 4]. Importantly, they are more frequently associated with neurofibromatosis type 1 (NF1) and tend to occur in younger individuals [4]. Although most studies showed a lack of significant prognostic differences between Amp-NENs and non-Amp-Duo-NENs, especially when only well-differentiated neuroendocrine tumors (NETs) were considered [3–10], Amp-NENs were shown to display a more aggressive behavior with a trend towards reduced overall survival in some studies [11–15]. In addition, there are several histologic differences between Amp-NENs and non-Amp-NENs. First, poorly differentiated neuroendocrine carcinomas (NECs) are more likely located in the ampullary region than in the non-ampullary duodenum [16]. Second, most ampullary NETs (Amp-NETs), arising either in the major ampulla or in the minor papilla, show a characteristic pseudo-glandular (tubulo-acinar) pattern, often with psammoma bodies, and extensive somatostatin expression, whereas the majority of non-ampullary duodenal NETs (non-Amp-Duo-NETs) show a typical nesting-to-trabecular architecture and are immunoreactive for gastrin and negative or only sparsely positive for somatostatin [16, 17]. Third, the so-called gangliocytic paraganglioma (GP), which the new 2022 World Health Organization (WHO) of Endocrine and Neuroendocrine Tumors has renamed composite gangliocytoma/neuroma and neuroendocrine tumor (CoGNET), exhibits a strong preference for major or minor ampullary regions [16–19]*.* Finally, treatment guidelines of duodenal NETs consider tumor site; indeed, surgical resection (local or radical) is generally indicated for Amp-NENs, regardless of the tumor size, while a fraction of small duodenal non-Amp-NETs may be treated endoscopically [1, 20, 21].

Despite these relevant differences, Amp-NENs and non-Amp-Duo-NENs are considered together by most studies evaluating the factors associated with metastatic potential and prognosis; thus, the current knowledge of Amp-NEN prognostic factors depends on limited, often single-institution, case series and case reports [22, 23] and/or national cancer databases with inherent well-known limitations [15]. The aim of this study was to analyze a large multicentric series of Amp-NENs, in order to identify specific prognostic factors.

## Materials and Methods

Pathology databases and Endocrine Tumor Registers of the Anatomic Pathology Departments of Pavia, “Vita-Salute San Raffaele” (Milan), Humanitas (Milan), Verona, Padua, Genoa, Insubria and Lausanne Universities, of Catholic University of Rome and of “Casa Sollievo della Sofferenza” of San Giovanni Rotondo were searched for cases diagnosed as “neuroendocrine tumor” or “neuroendocrine carcinoma” or “endocrine tumor” or “endocrine carcinoma” or “carcinoid” or “gangliocytic paraganglioma” of the major or minor papilla/ampulla between 1987 and 2020.

Cases included in this study fulfilled the following criteria: (a) NEN with tumor epicenter, i.e., the major portion of the tumor, predominantly situated in the major or minor ampulla at gross examination/dissection of pancreatoduodenectomy specimens or, alternatively, NEN found in ampullectomy specimens with preoperative and intraoperative evidence of NEN epicenter within the major (or minor) ampulla; (b) histologic presence of pancreatobiliary-type ampullary ducts adjacent to NEN. Primary pancreatic NENs, non-Amp-Duo-NENs with extension to the major or minor ampulla regions, and mixed neuroendocrine-non-neuroendocrine neoplasms (MiNENs) were excluded.

All clinical (patient age at diagnosis, tumor site, hyperfunctional endocrine syndrome, hereditary cancer syndrome, type of resection, presence and site of distant metastasis) and histopathologic data were recorded. In particular, full somatostatinoma syndrome was diagnosed when at least three of the following features were present: recent onset diabetes mellitus, noticeably increased plasma and/or tumor somatostatin, anemia, reduced gastric acid secretion, bile stones, diarrhea/steatorrhea, and loss of weight [24, 25]. All relevant clinical, endoscopic, imaging, and serologic data were obtained using hospital clinical charts, local tumor registries, and contacts with general practitioners. All follow-up information was noted.

Histologically, the following parameters were recorded: tumor differentiation, histologic subtype, mitotic index per 2 mm^2^, proliferation index using Ki67, vascular invasion in small (lymphatics, capillaries, or post-capillary venules) and large vessels, tumor necrosis, tumor size, level of invasion, the total number of isolated loco-regional lymph nodes, and the number of metastases in loco-regional nodes (lymph node metastases—LNMs). All slides were stained with hematoxylin and eosin (H&E) for morphologic evaluation; immunohistochemistry for synaptophysin (monoclonal, clone DAK-SYNAP, Dako, Carpinteria, CA) was used to confirm neuroendocrine differentiation, for Ki67 (monoclonal, clone MIB1; Dako) to assign WHO grade, and for CD31 (clone JC70A, Dako) to identify invasion in small vessels. All available slides were reviewed or, when not available, staining was performed. Large vein involvement was distinguished from small vessel invasion when a smooth muscle layer and/or elastic lamina were identified. In addition, immunohistochemistry for somatostatin (polyclonal, Dako) was carried out in Amp-NETs for histologic subtyping. Gastrin (polyclonal; Dako) and ACTH (clone 02A3, Dako) immunostains were performed in functioning NETs with Zollinger-Ellison and Cushing syndrome, respectively, while non-functioning conventional NETs were also tested for gastrin (polyclonal, Dako), serotonin (polyclonal; Novocastra, Newcastle, UK), and pancreatic polypeptide (polyclonal; Peninsula Laboratories, Belmont, CA). In cases identified as GP/CoGNET, S100 immunohistochemistry (polyclonal, Dako) was also performed to highlight sustentacular/Schwannian components.

Neoplasms were diagnosed as large cell NEC when showing solid, irregular poorly formed nests and trabeculae of large cells with atypia, vesicular nuclei, and evident nucleoli. Small cell NEC was diagnosed when tumors showed diffuse, solid sheets of small-to-intermediate-sized atypical cells with scant cytoplasm and round or spindle morphology. High-grade carcinomas were also frequently associated with necrotic foci and nuclear streaming artifacts, as well as desmoplastic stroma. NETs were diagnosed as such when well-differentiated morphology was seen [25]. According to WHO 2019 criteria, grade in NETs was assigned according to Ki67 index and mitotic count, while NECs were considered high-grade (grade 3) by definition. The proliferation labeling index with Ki67 was assessed following the European Neuroendocrine Tumor Society (ENETS)/WHO recommendations. In particular, after identifying the hot spot area of highest nuclear labeling, the percentage of immunostained tumor cells/neoplastic cells (at least 500 cells) was evaluated manually on printed high magnification (× 400) microphotographs of the hot spots. Tumors were diagnosed as G1 when mitotic index was < 2 mitoses/2 mm^2^ and proliferation index was < 3% Ki67 index; tumors were diagnosed as G2 when mitotic index was between 2 and 20 mitoses/2 mm^2^ or proliferation index as between 3 and 20% Ki67 index; G3 NETs were diagnosed as such when mitotic index was greater than 20 mitoses/2 mm^2^ or Ki67 > 20%. If NENs showed Ki67 labeling index > 20%, these were re-assessed for histological differentiation using the reproducible morphologic criteria recently proposed by Elvebakken et al., which include tumor architecture (organoid in NETs vs non-organoid or diffuse for NECs), stroma (hyalinized in NETs vs desmoplastic in NECs), and capillary network (vessels in direct contact with tumor cells in NETs or more distant in NECs) [26].

Amp-NETs were histologically sub-classified into (i) GP/CoGNET, defined by the typical triphasic morphology (paraganglioid, Schwannian-like and ganglion cell-like components); (ii) ampullary-type somatostatin-producing D cell NETs (SOM-NETs), characterized by extensive (> 50%) somatostatin expression by tumor cells and at least focal pseudo-glandular structure, with or without psammoma bodies; and (iii) conventional NETs, i.e., the remaining cases which do not fulfill the criteria for GP or SOM-NET [16, 17, 25].

The neoplasms were staged according to the 8th American Joint Committee on Cancer (AJCC) TNM staging system, which is different for Amp-NETs and Amp-NECs, as the latter are staged with the same TNM system as ampullary adenocarcinomas [27]. Minor papilla-ampulla NETs were staged using the same criteria adopted by AJCC for major ampulla NETs, as following: pT1 (tumor dimension ≤ 1 cm and confined within the sphincter), pT2 (tumor invades through sphincter into duodenal submucosa or muscularis propria, or is > 1 cm), pT3 (tumor invades the pancreas or peripancreatic adipose tissue), and pT4 (tumor invades the visceral peritoneum (serosa) or other organs). N stage was assigned only in cases which underwent surgical lymphadenectomy (pN stage); radiological N stage was not assigned in consideration of the high likelihood of understaging with imaging/preoperative techniques observed in this setting [28].

Following the AJCC criteria for ampullary adenocarcinomas, pN stage was subdivided into pN0 (no local LNM), pN1 (1–3 LNMs), and pN2 (> 3 LNMs) for both Amp-NETs and Amp-NECs. In addition, lymph node ratio, defined as the number of positive lymph nodes over the total number of isolated nodes, was calculated in N + cases. Despite the known limitations, for assessment of distant metastases at diagnosis, computed tomography (CT) imaging and/or ^68^Ga-labeled tetraazacyclododecane tetraacetic acid (DOTA)–peptide positron emission tomography (PET)/CT scans were also considered (when available).

Two surgical pathologists specialized in gastrointestinal and neuroendocrine pathology (AV and ES) performed central pathology review.

The study was performed in agreement with the clinical standards laid down in the 1975 Declaration of Helsinki and its revision and was approved by the ethics committee of Pavia (protocol number: 20210027824).

## Statistical Analysis

This is a longitudinal retrospective study. Descriptive statistics were computed as median and 25th–75th percentiles for continuous variables and as counts and percentages for categorical variables. The Mann–Whitney test was used to compare continuous variables between neoplasm types while the Fisher’s exact test (or χ2 test) was used to compare categorical variables. Logistic regression was used to assess the association of a series of tumor characteristics and the presence of N + disease only in NET cases which underwent lymphadenectomy. In case of 0 events in one category, an exact logistic model was fitted. Odds ratios (OR) and 95% confidence intervals (CI) were computed. Variables with a *p* < 0.1 on univariable analysis were included in a multivariable model. Model discrimination was measured using the model area under the receiver operating characteristic (ROC) curve (and 95%CI). The optimal size cutoff for predicting LNMs in NETs was identified by ROC curve analysis. Sensitivity, specificity, area under the ROC curve, and 95% CI were computed. Follow-up was computed from the date of diagnosis to the date of death or last available follow-up for censored patients. The reverse Kaplan–Meier method was computed by means of the median follow-up and its interquartile range (25th–75th) was computed by means. Disease-specific survival (DSS) was calculated. Kaplan–Meier cumulative survival curves were plotted and compared with the log-rank test. Cox regression analysis assessed the strength of association between candidate risk factors and mortality. Hazard ratios (HR) and 95% CI were derived from the models. The proportional hazard assumption was tested based on Schoenfeld residuals. Due to the low number of disease-related deaths, only univariable analysis was possible. Intra-center correlation was accounted for by computing Huber-White robust standard errors while clustering on center for all the fitted models. A two-sided *p* value < 0.05 was considered statistically significant. For post hoc comparisons, Bonferroni correction is applied. Stata 17 (StataCorp, College Station, TX, USA) was used for computation.

## Results

### Prevalence and Clinico-pathologic Features of Amp-NETs and Amp-NECs

**Fig. 1:**
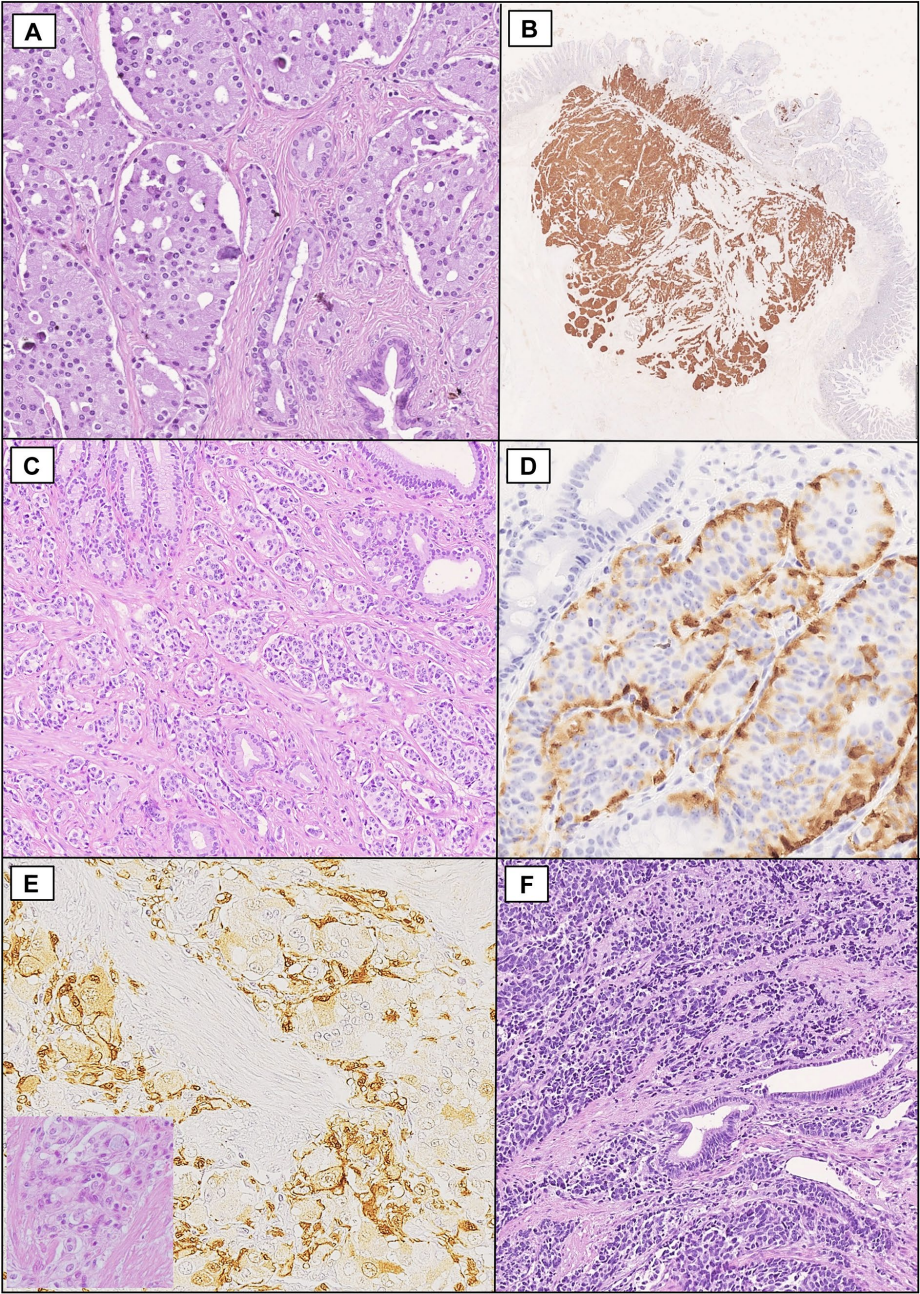
Histologic types of ampullary neuroendocrine neoplasms. **A-B** A somatostatin-producing neuroendocrine tumor (SOM-NET) of the major ampulla, with tubulo-glandular architecture and psammoma bodies. Note in (**A**), on the lower right, the presence of residual ampullary ductules and in (**B**) the extensive and strong somatostatin expression by tumor cells (**A**, hematoxylin and eosin; **B**, somatostatin immunohistochemistry). **C-D** An unusual case of ACTH-producing ampullary NET associated with Cushing syndrome, showing with a conventional, nested-to-trabecular structure and ACTH expression by many tumor cells (**C**, hematoxylin and eosin; **D**, ACTH immunohistochemistry). **E** A gangliocytic paraganglioma (GP)/composite gangliocytoma/neuroma and neuroendocrine tumor (CoGNET) showing a S100-negative paraganglioid component, with epithelioid cells arranged in solid nests (see the inlet, hematoxylin and eosin), rare ganglion-like cells, and S100-positive sustentacular and stromal cells (S100 immunohistochemistry). **F** A small cell type neuroendocrine carcinoma (NEC) of the major ampulla; note, in the center, some residual ampullary ductules

The present series is composed of 119 Amp-NENs, which included 93 (78%) Amp-NETs and 26 (22%) Amp-NECs (Fig. 1). Amp-NETs were graded as G1 (67 cases, 73%) and G2 (25 cases, 27%) NETs, while the WHO grade could not be reliably assessed in one case for the lack of an additional tumor section to perform Ki67 immunohistochemistry. Interestingly, no NET G3 was identified. Regarding histologic NET subtypes, 58 (62%) cases were SOM-NETs, characterized by extensive somatostatin expression and tubulo-acinar structure, only one of which (2%) was associated with a full-blown somatostatinoma syndrome, 8 (9%) were GPs/CoGNETs, and 27 (29%) were conventional NETs. The latter comprised 24 non-functioning NETs and three functioning NETs, which were two gastrinomas associated with Zollinger-Ellison syndrome and one ACTH-producing NET associated with Cushing syndrome. In addition, immunohistochemical expression of hormones in non-functioning conventional NETs was always focal, involving less than 10% of neoplastic cells, and relatively rare (gastrin in 5 cases, somatostatin in 4 cases, serotonin in 1 case, and pancreatic polypeptide in 2 cases). SOM-NET patients were significantly younger (median age: 55 years; 25th–75th: 44–63) than those with conventional NETs (59 years; 25th–75th: 49.5–70, *p* = 0.048) and more frequently associated with hereditary tumor syndromes (23% vs 3%, *p* = 0.029), in particular with NF1. In addition, SOM-NETs were significantly larger in size (median size: 20 mm, 25th–75th: 14.25–29.5) in comparison with conventional NETs (10 mm, 25th–75th: 7–19.5; *p* value: 0.003). Amp-NECs comprised 14 “small cell subtype,” while the remaining 12 were large cell NECs.

According to AJCC criteria, pT stage could be assigned to 88 Amp-NETs (pT1: 11 cases, 12.5%, pT2: 45 cases, 51.1%, pT3: 32 cases, 36.4%) and to 20 NECs, most of which (70%) were pT3, while 4 (20%) were pT2 and two (10%) pT1. No pT4 case was observed. AJCC TNM stage at diagnosis was assigned to 62 Amp-NETs (stage I: 6 cases, 9.7%; stage II: 9 cases, 14.5%, stage III: 38, 61.3%, stage IV: 9, 14.5%) and to 23 NECs (stage I: 0%, stage II: 1 case (4.3%), stage III: 12 cases, 52.2%, stage IV: 10 cases, 43.5%).

A detailed comparison between clinico-pathologic features of Amp-NETs and Amp-NECs is outlined in Table 1. Amp-NEC patients were significantly older (median age: 66 years) in comparison with Amp-NET patients (median age: 57 years, *p* = 0.0019) and showed a stronger predominance for male gender (77% vs 52%, *p* = 0.025). Amp-NETs feature a higher association with hereditary tumor predisposition syndromes (15% vs 4%), although the difference was not statistically significant. In particular, 12 NETs (all of which categorized as SOM-NETs and three of which with double, synchronous tumors involving both the major and minor ampulla) were NF1-associated, two NETs were MEN1-associated, whereas one NEC arose in the setting of classic MEN2A syndrome (in a patient with a history of medullary thyroid carcinoma and primary hyperparathyroidism). Regarding the type of treatment, most patients in both differentiation groups underwent pancreatoduodenectomy; however, about a quarter of Amp-NETs were treated with local resection (such as ampullectomy), which was never performed in NECs. Although the vast majority of both Amp-NETs and Amp-NECs were detected in the major ampulla region, a small proportion (16 cases, 17%) of NETs only arose in the minor papilla/ampulla. As expected, the median number of mitoses per 2 mm^2^ and the median Ki67 proliferative index were significantly higher in NECs (median mitotic rate: 32; median Ki67: 70%), compared to NETs, which showed a very low proliferative activity as a group (median mitotic rate: 0.5; median Ki67: 1.5%). In addition, NECs showed a significantly larger size (median: 25 mm) compared to NETs (median. 16 mm, *p* = 0.01), a higher proportion of invasion of the pancreas or peripancreatic soft tissues (46% vs 34%) and of distant metastases at diagnosis (42% vs 11%), mostly to the liver, as reflected by the higher percentages of pT3 and stage IV observed in Amp-NECs. Nevertheless, a direct comparison of AJCC pT stages between Amp-NETs and Amp-NECs is not possible because of some differences in TNM staging criteria adopted by AJCC for the two disease types. Tumor necrosis was found in 88% of Amp-NECs whereas no Amp-NETs showed evidence of tumor necrosis.

**Table 1:** Clinico-pathologic features of 119 Amp-NEN cases according to histologic differentiation

**Variable**	**Amp-NETs**	**Amp-NECs**	***p* value**
**Total N. of cases (% of total Amp-NENs)**	93 (78%)	26 (22%)	
**Patient age at diagnosis, years, median (25th–75th)**	57 (47–66)	66 (60–71)	**0.002**
**Patient age at diagnosis > 60 years,** *N* (%)	32 (34)	19 (73)	**0.001**
**Male gender,** *N* (%)	48 (52)	20 (77)	**0.025**
**Hereditary tumor syndrome, *N* (%)**	14 (15)	1 (4)	**0.186**
**Functionality, *N* (%)**	4 (4)	0	**0.575**
**Type of treatment, *N* (%)**			**< 0.001**
Local resection (ampullectomy or transduodenal excision)	22 (24)	0	
Pancreatoduodenectomy	65 (70)	19 (73)	
Biopsy + medical treatment	1 (1)	7 (27)	
Unknown	5 (5)	0	
**Tumor site, *N* (%)**			**0.021**
Major ampulla	77 (83)	26 (100)	
Minor ampulla	16 (17)	0	
**Tumor size, mm, median (25th–75th)**	16 (10–25)	25 (20–30)	**0.010**
**Mitotic rate, number/2 mm**^**2**^**, median (25th–75th)**	0.5 (0–1)	32 (20–52)	**< 0.001**
**Ki67 proliferative index, %, median (25th–75th)**	1.5 (1–2)	70 (60–74)	**< 0.001**
**Small vessel invasion, *N* (%)**	51 (55)	20 (77)	**< 0.001**
**Level of invasion, *N* (%)**			**0.002**
Within the muscle sphincter	12 (13)	0	
Duodenal submucosa or muscularis propria	44 (47)	7 (27)	
Pancreas or peripancreatic tissues	32 (35)	12 (46)	
Unknown	5 (5)	7 (27)	
LNM, *N* (%)^*^	44 (75)	17 (89)	**0.216**
pN, *N* (%)^*^			**0.179**
pN0	16 (27)	2 (11)	
pN1 (1–3 LNM)	19 (32)	10 (52)	
pN2 (> 3 LNM)	24 (41)	7 (37)	
Distant metastases at diagnosis, *N* (%)^**^	9 (11)	10 (42)	**0.001**
**Disease-specific mortality rate** per 100 person-year (95% CI)	0.87 (0.39–1.95)	53.63 (34.21–84.07)	**< 0.001**

*Amp-NET* ampullary neuroendocrine tumor, *Amp-NEC* ampullary neuroendocrine carcinoma, *Amp-NEN* ampullary neuroendocrine neoplasm, *CI* confidence interval, *LNM* lymph node metastasis

^*^Fifty-nine Amp-NET patients and 19 Amp-NEC patients underwent lymphadenectomy with histologic lymph node assessment; ^**^clinical and/or pathologic data on distant metastasis at diagnosis were available for 84 Amp-NET patients and 24 Amp-NEC patients

Finally, we found a higher rate of small vessel invasion in NECs compared to NETs (77% vs 55%, *p* < 0.001), despite no significant difference in the LNM rate or pN stage between the two groups. While a half of NECs showed both small and large vessel angioinvasion, only small vessel invasion was identified in NETs. Moreover, lymph node ratio in cases with LNMs was similar between Amp-NETs (median 0.3, 25th–75th: 0.11–0.40) and Amp-NECs (0.20, 0.10–0.29, *p* = 0.389), as well as was the total number of lymph nodes examined (median: 17, 25th–75th: 9–25 for NETs vs 16: 10–19 for NECs, *p* = 0.545).

**Fig. 2:**
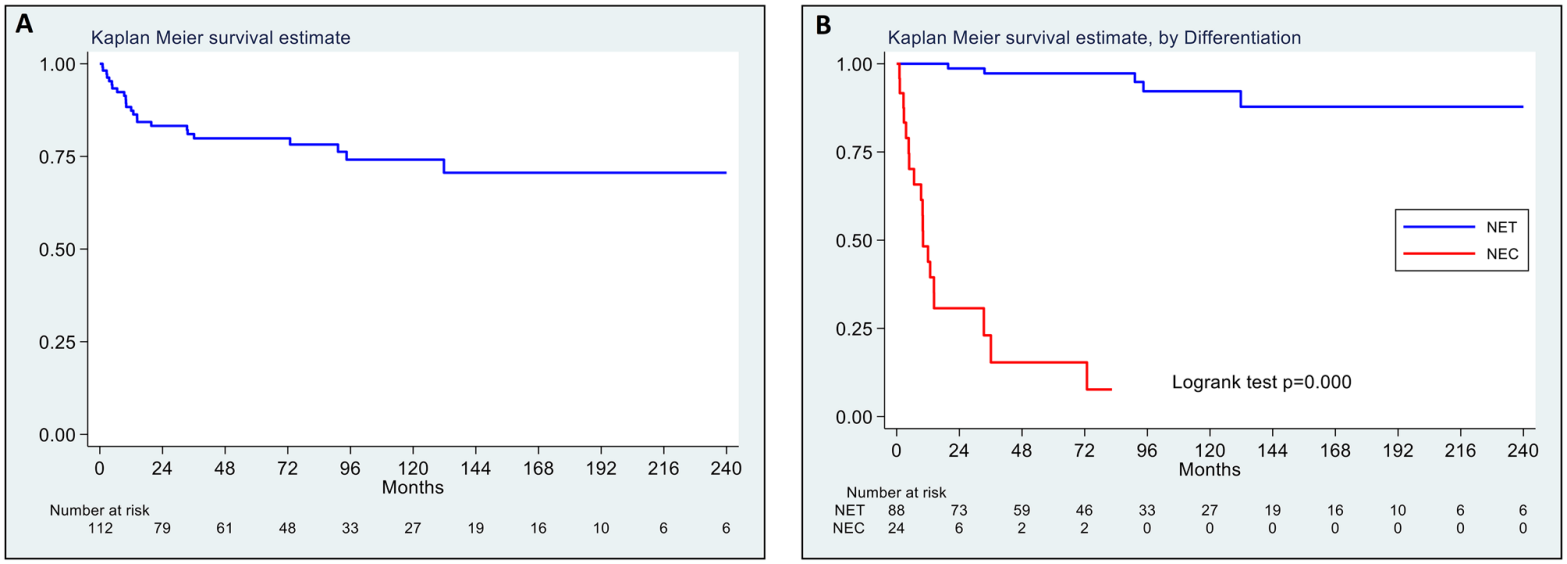
Kaplan–Meier disease-specific survival estimates of the entire cohort of 112 patients ampullary neuroendocrine neoplasms (Amp-NENs) (**A**); Kaplan–Meier disease-specific survival estimates by histologic differentiation, i.e., well-differentiated neuroendocrine tumors (NETs) versus poorly differentiated neuroendocrine carcinomas (NECs) (**B**)

Median follow-up for the 112 patients (88 with Amp-NETs and 24 with Amp-NECs) was 78 months (25th–75th: 38–142). Neoplasm-related death was observed in 19 Amp-NEC patients (estimated median survival: 10 months) and in 6 Amp-NET patients (median survival: not reached). Tumor differentiation was a strong prognostic factor in Amp-NENs according to disease-specific survival analysis which showed that NEC patients showed significantly worse outcome (HR: 80.74, 95% CI: 32.04–203.45) in comparison with NET patients (Fig. 2). Given the clear prognostic difference between Amp-NETs and Amp-NECs and the limited number of Amp-NECs under investigation, subgroup analysis in Amp-NETs and Amp-NECs was performed to look for potential predictors of LNMs and/or prognostic factors. No statistically significant prognostic factor was identified in Amp-NEC subgroup analysis, while the Amp-NET subgroup analysis is detailed below.

### Predictors of LNMs in Amp-NETs

Among 59 Amp-NET patients who underwent lymphadenectomy with lymph node histologic examination, the following factors were significantly associated with LNMs: major ampulla tumor site, WHO tumor grade G2, SOM-NET histologic subtype, small vessel invasion, tumor size, pancreatic invasion, and pT stage (Table 2). These parameters remained significantly associated with LNMs also when 34 NET patients with negative preoperative imaging lymph node assessment who did not receive lymphadenectomy were added to Amp-NETs with pathologically confirmed negative loco-regional lymph nodes. In our cohort, the optimal empirical cutoff for tumor size in predicting LNMs in Amp-NETs was 16 mm (area under ROC curve: 0.71; sensitivity: 68%, specificity: 73%; positive predictive value: 88.2% (72.5–96.7%); negative predictive value: 44% (24.4–65.1%). In a multivariable model including non-collinear variables with *p* < 0.1, tumor site, small vessel invasion, and tumor size (> 16 mm vs ≤ 16 mm) remained significantly associated with LNMs (Table 3). The model performed well, with an area under the ROC curve of 0.89.

**Table 2:** Predictors of lymph node metastasis in NET patients with lymphadenectomy (*n* = 59) at univariable analysis

**Variable**	**Lymph node metastasis, *N* (%)**		**OR (95% CI)**	***p* value (logistic regression)**
	**Present**	**Absent**		
**Patient age at diagnosis**				
≤ 60 years	31 (70)	7 (47)	1	0.111
> 60 years	13 (30)	8 (53)	0.37 (0.11–1.26)	
**Patient gender**				
Male	25 (57)	6 (40)	1	0.064
Female	19 (43)	9 (60)	0.51 (0.24–1.09)	
**Tumor site**				
Major ampulla	40 (91)	9 (60)	1	**0.003**
Minor ampulla	4 (9)	6 (40)	0.15 (0.04–0.53)	
**Hereditary tumor syndrome**				
No	39 (89)	11 (73)	1	0.231
Yes	5 (11)	4 (27)	0.35 (0.06–1.94)	
**WHO tumor grade**				
** G1**	27 (61)	13 (89)	1	**0.027**
** G2**	17 (39)	2 (13)	4.09 (1.17–14.31)	
**Histologic Subtype**				**0.016**
SOM-NET	29 (66)	8 (53)	1	
CoGNET	1 (2)	1 (7)	0.28 (0.12–0.65)	**0.003**
Conventional NET	14 (32)	6 (40)	0.64 (0.11–3.94)	0.634
Small vessel invasion^*^				
No	8 (20)	11 (73)	1	**0.001**
Yes	35 (80)	4 (27)	12.03 (2.92–49.64)	
**Tumor size (above or below the median)**				
≤ 16 mm	14 (32)	11 (73)	1	**< 0.001**
> 16 mm	30 (68)	4 (27)	5.89 (2.89–12.04)	
**Tumor size (three groups)**				**0.001**
≤ 10 mm	8 (18)	7 (47)	1	
11–20 mm	15 (34)	6 (40)	2.19 (0.40–12.01)	0.368
> 20 mm	21 (48)	2 (13)	9.19 (2.74–30.77)	**< 0.001**
Pancreatic invasion^*^				
Yes	25 (58)	2 (13)	9.03 (2.84–28.74)	**< 0.001**
No	18 (42)	13 (87)	1	
pT stage^**^				**< 0.001**
pT1	0	6 (40)	1	
pT2	18 (41)	7 (47)	11.13 (1.24– + inf)	**0.030**
pT3	26 (59)	2 (13)	36.13 (3.90– + inf)	**< 0.001**

*CoGNET* composite gangliocytoma/neuroma and neuroendocrine tumor, *NET* neuroendocrine tumor, *SOM-NET* somatostatin-expressing D cell neuroendocrine tumor

^*^Unknown in one case; ^**^exact logistic regression fitted

**Table 3:** Multivariable model for lymph node metastasis in NETs with lymphadenectomy (including non-collinear variable with *p* < 0.1)

**Variable**	**OR (95% CI)**	***p* value**
**Gender (female vs male)**	1.01 (0.19–5.24)	0.993
**Tumor site (minor vs major ampulla)**	0.14 (0.03–0.73)	**0.019**
**WHO grade (G2 vs G1)**	1.01 (0.08–12.57)	0.994
**Small vessel invasion (yes vs no)**	24.59 (5.78–104.68)	**< 0.001**
**Tumor size (> 16 mm vs < = 16 mm)**	6.55 (1.09–39.21)	**0.040**

Model: *p* < 0.001; ROC area: 0.89, 95% CI: 0.77–0.95

### Predictors of Cancer-Specific Survival in Amp-NETs

**Fig. 3:**
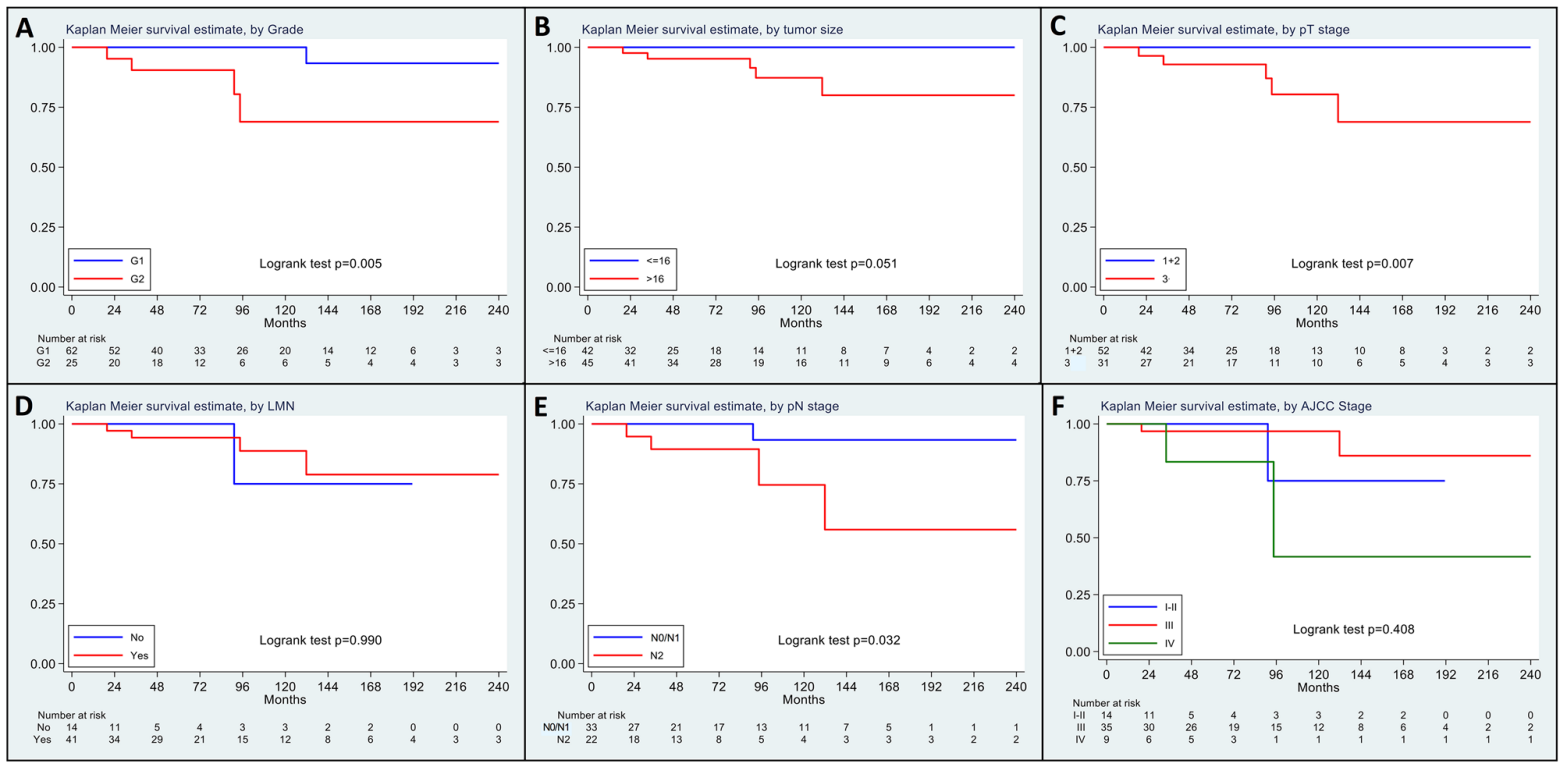
Kaplan–Meier disease-specific survival estimates in patients with ampullary neuroendocrine tumors (Amp-NETs) by WHO histologic grade (**A**); by tumor size above or below the median (16 mm) (**B**); by T stage (**C**); by presence of lymph node metastasis (LNM) (**D**); by number of LNMs (pN2: > 3 LNMs) (**E**); by AJCC stage (**F**)

Eighty-eight Amp-NETs patients with complete follow-up data entered disease-specific survival analysis. Factors significantly associated with worse survival in Amp-NET patients were (i) patient age at diagnosis > 60 years, (ii) WHO tumor grade G2, (iii) small vessel invasion, (iv) pancreatic invasion (pT3), and (v) distant metastasis at diagnosis (Table 4). No significant prognostic difference was found between SOM-NET and conventional NET patients (*p* = 0.144), while GP/CoGNET cases did not show tumor-related deaths. Although the presence of LNM and lymph node ratio (above the median) were not related to worse patient outcomes, we found that cases with more than three LNMs (i.e., pN2 stage) showed significantly higher mortality rates compared to the remaining cases (Fig. 3). The rate of pN2 cases was significantly (*p* = 0.026) higher when at least 12 lymph nodes were examined (20/39, 51%) compared to those with a total number of lymph nodes < 12 (4/20, 20%), suggesting that lack of adequate lymph node harvesting could lead to Amp-NET understaging.

**Table 4:** Predictors of disease-specific survival in well-differentiated Amp-NET patients with follow-up (*n* = 88)

**Variable**	**Rate of death per 100 person-year (95% CI)**	**HR (95% CI)**	***p* value**
**Patient age at diagnosis**			
≤ 60 years	0.57 (0.18–1.76)	1	** < 0.001**
> 60 years	1.84 (0.59–5.70)	4.51 (2.25–9.04)	
**Patient gender**			
Male	0.94 (0.30–2.91)	1	0.3612
Female	0.81 (0.26–2.51)	0.77 (0.43–1.36)	
**Tumor site**			
Major ampulla	1.04 (0.47–2.31)	NE	0.3021^*^
Minor ampulla	0		
**Hereditary tumor syndrome**			
No	0.86 (0.36–2.07)	1	0.9778
Yes	0.90 (0.13–6.43)	1.04 (0.09–11.99)	
**Functionality**			
Yes	0	NE	0.770^*^
No	0.89 (0.40–1.98)		
**Type of resection**			
Local	0	NE	0.1025^*^
Pancreatoduodenectomy	1.33 (0.60–2.97)		
**WHO tumor grade**			
G1	0.21 (0.03–1.47)	1	** < 0.001**
G2	2.56 (1.07–6.15)	12.61 (6.41–24.80)	
**Histologic subtype**			0.364^*^
SOM-NET	0.81 (0.30–2.15)	1	
Conventional NET	1.70 (0.43–6.79)	2.69 (0.71–10.18)	0.144
CoGNET	0	NE	
**Small vessel invasion**			
No	0.32 (0.04–2.25)	1	**0.004**
Yes	1.46 (0.61–3.51)	4.13 (1.42–12.00)	
**Tumor size (median)**			
> 16 mm	0	NE	0.051^*^
≤ 16 mm	1.50 (0.67–3.34)		
**Tumor size (three groups)**			0.176^*^
≤ 10 mm	0	NE	
11–20 mm	0.42 (0.06–2.99)		
> 20 mm	1.70 (0.71–4.08)		
**Pancreatic invasion**			
Yes (pT3)	1.81 (0.75–4.34)	NE	0.007^*^
No (pT1–pT2)	0		
LNM^**^			
Absent	1.35 (0.19–9–56)	1	1.000
Present	1.59 (0.66–3.82)	0.99 (0.32–3.06)	
Lymph node ratio^**^			
≤ 0.3 (median)	1.41 (0.35–5.63)	1	0.860
> 0.3 (median)	1.74 (0.56–5.39)	0.85 (0.14–5.03)	
Number of LNM (pN)^**^			
pN0-1	0.42 (0.06–2.97)	1	**0.004**
pN2	3.34 (1.39–8.01)	7.64 (1.93–30.24)	
**Distant metastasis at dgn**			
Absent	0.67 (0.25–1.79)	1	**0.005**
Present	3.60 (0.90–14.40)	4.89 (1.61–14.88)	
**AJCC stage**			0.408^*^
I–II	1.35 (0.19–9.56)	1	
III	1.06 (0.34–3.29)	0.70 (0.37–1.30)	0.766
IV	3.60 (0.90–14.40)	2.32 (0.63–8.57)	0.618

*CoGNET* composite gangliocytoma/neuroma and neuroendocrine tumor, *dgn* diagnosis, *LNM* lymph node metastasis, *NE* not estimable, *NET* neuroendocrine tumor, *SOM-NET* somatostatin-expressing D cell neuroendocrine tumor

^*^p value calculated with Log-rank test; ^**^no lymphadenectomy in 32 patients. Five patients were lost to follow-up

## Discussion

In the present study of a large multicentric series of Amp-NENs, classification by histologic differentiation highlighted marked clinico-pathologic differences between well-differentiated Amp-NETs (78% of cases) and poorly differentiated Amp-NECs (22% of cases). First, Amp-NETs arose in significantly younger patients (median age: 57 years) compared to Amp-NECs (66 years) and are more frequently associated with hereditary tumor syndromes (15% versus 4%), in particular with NF1, which we observed in 13% of Amp-NETs as a whole and in 21% of SOM-NETs in our series [24, 25]. Second, Amp-NECs were always found in the region of major ampulla, while 17% of Amp-NETs were found in the minor papilla/ampulla. Third, Amp-NECs were larger (median size: 25 mm) than Amp-NETs (median size: 16 mm), as already reported by Albores-Saavedra et al. using data from the National Cancer Institute’s Surveillance, Epidemiology and End Results (SEER) Program [29]. In addition, the frequency of adverse histologic features, such as small and large vessel invasion and deep level of invasion, as well as the rate of distant metastasis at diagnosis, was significantly higher in Amp-NEC than in Amp-NET cases. Interestingly, although Albores-Saavedra et al. found a lower rate of lymph node involvement in Amp-NETs compared to Amp-NECs, in our series, the frequency of LNMs in patients who underwent lymphadenectomy was very high for both neoplastic entities (75% for Amp-NETs and 89% for Amp-NECs), without significant differences between them. Finally, as expected, the disease-specific survival was tremendously worse in Amp-NEC compared to Amp-NET patients. In fact, Amp-NECs are associated with a generally ominous prognosis, like NECs of other gastroenteropancreatic sites [30–32]. Altogether, these findings support the pivotal importance of distinguishing NECs from NETs also in ampullary regions. This histologic separation is usually straightforward as, among Amp-NETs, NETs G3 were exceptionally identified, as observed in the present investigation, as well as by other authors [33]. Whether peculiar molecular features may underline the extreme rarity of NETs G3 in the ampullary region, as well as in other sites, such as appendix, presacral region, breast, and prostate, is currently unknown.

Importantly, we identified prognostic factors specific for Amp-NET patients, which were largely unknown, due to the rarity of such tumors, while no prognostic factor could be identified in Amp-NECs, likely related to the small number of cases.

The following factors were associated with worse disease-specific survival in Amp-NETs: (i) patient age at diagnosis > 60 years, (ii) WHO tumor grade G2, (iii) small vessel invasion, (iv) pancreatic invasion, and (v) distant metastasis. The limited number of tumor-related deaths (*n* = 6) prevented multivariable analysis. We found, for the first time, that WHO tumor grade (based on mitotic index and Ki67 proliferative index) proved to be a strong predictor of survival (HR: 12.61, *p* < 0.001 for G2 vs G1 tumors), as demonstrated in pancreatic and other gastrointestinal NETs [34]. In a recent investigation on Amp-NETs based on National Cancer Database, Ruff et al. found that age, Charlson–Deyo score of 2 or C3, “grade 2” or “grade 3” tumors, and metastatic disease were associated with decreased survival on multivariable analysis [15]. However, a direct comparison of their findings on tumor grade with our study based on WHO 2019 grading system is not feasible because assessment of Ki67 proliferative index was not recorded in the National Cancer Database and at least a proportion of their “grade 3 tumors” might be Amp-NECs. Interestingly, Ruff et al. reported that median overall survival was significantly worse for patients resected for Amp-NETs (122 months) compared with non-Amp-Duo-NETs (145 months) and pancreatic head NETs (132 months), confirming the relatively more aggressive biology of Amp-NETs [15].

The prognostic impact of vascular invasion has been previously reported in duodenal-ampullary NETs [8, 13, 16] and it could impact treatment decisions. Small vessel invasion, as assessed in our investigation, includes both lymphatic and blood vessel invasion [35]; whether separation of angioinvasion from lymphatic invasion improves prognostic stratification in this setting requires further investigations.

On the contrary, the presence of LNMs in lymphadenectomy specimens was not significantly associated with patient outcome. The limited, if any, prognostic impact of LNMs on patient survival in Amp-NETs has been hypothesized in previous investigations on smaller series or national databases [15, 36–38] or in studies including both Amp-NENs and non-Amp-Duo-NENs without subgroup analysis [3–6, 12, 13, 16]. However, the lack of prognostic relevance of LNMs might reflect a therapeutic effect of radical lymphadenectomy [13]. Therefore, additional studies are needed to confirm that LNMs do not affect prognosis even when not surgically treated. Interestingly, we found that patients with > 3 LNMs (i.e., pN2) showed a worse outcome, suggesting that a three-tiered pN substaging based on the number of LNMs, similar to that applied to ampullary adenocarcinoma and Amp-NECs, might be prognostically useful also in Amp-NET patients. This finding is also in keeping with recent observations in pancreatic or small bowel NETs, showing that patients with N +  ≥ 4 metastatic nodes had a worse recurrence-free survival compared to N + patients with 1 to 3 nodal metastases or N0 [39–41].

Histologically, most (62%) Amp-NETs belong to SOM-NET subtype (a histologic subtype with strong and selective preference for the major/minor ampullary regions, where it is very rarely, if ever, associated with a full-blown clinical somatostatinoma syndrome), followed by conventional NETs (29%), a fraction (11%) of which are associated with a hyperfunctioning syndrome (two gastrinomas associated with Zollinger-Ellison syndrome and one ACTH-producing NET associated with Cushing syndrome). No prognostic value of histologic subtyping emerged from the present investigation; however, we can confirm that the rare GPs/CoGNETs are less frequently associated with LNMs in comparison with SOM-NETs and they behave in a very indolent fashion, as previously suggested [16, 18, 24]. Moreover, in the rare LNMs by GPs, both the epithelial NET component and the ganglioneuroma component may be found, supporting the nomenclature change to CoGNET of this neoplastic lesion as endorsed by the 2022 WHO Classification of Endocrine and Neuroendocrine Tumors [16, 19].

Previous studies have suggested that Amp-NETs tumor size has no prognostic implication and no relationship with metastases [1, 5, 11, 42–44], as even very small (< 1 cm) Amp-NETs may metastasize. However, in some studies, tumor size > 2 cm was associated with higher tumor recurrence or worse patient prognosis [22, 23, 37]. In our study, 50% of Amp-NETs ≤ 1 cm with lymphadenectomy revealed LNMs and tumor size was not related to patient survival even using different cutoff values. We found that the best empirical size cutoff for predicting LNM in Amp-NETs was 16 mm, with an acceptable positive predictive value (88%), despite a too low negative predictive value (44%), indicating, once again, that tumor size alone is not enough to reliably predict Amp-NET aggressiveness.

As upfront surgery for NECs has not shown clear survival benefits [45], its role is still controversial, even when tumors are localized at diagnosis. On the other hand, the current ENETS guidelines [1, 46] promote surgical resection in resectable Amp-NETs, regardless of tumor size. Indeed, such cases should be discussed after expert histologic evaluation/revision and assessment of tumor size, level of invasion and regional lymph nodes by including endoscopic ultrasonography (EUS), and whole-body imaging modalities [20, 21]. To date, lymphadenectomy is as a rule performed when LNMs are preoperatively known or detected intraoperatively [23, 24]. As we found that small vessel invasion and tumor size > 16 mm were factors independently associated with LNMs at multivariable analysis, we suggest that lymphadenectomy should be performed when these features are present. Regarding the extent of surgical resection (local versus radical), 2020 French guidelines state that local resection (in expert centers) might be sufficient for small Amp-NETs without evidence of LNMs, especially in patients with high surgical risk factors [21]. Our findings may support such an approach with some refinements, indicating that local surgical resection (such as ampullectomy or transduodenal tumor excision) may be an option that could be judiciously considered in very selected cases when preoperative biopsy reveals a small, G1 Amp-NET without small vessel invasion and EUS excludes pancreatic invasion and/or the patient is unfit for radical surgery. Interestingly, minor ampulla NETs were significantly less associated with LNMs and none of them caused patient death; therefore, a less aggressive surgical approach should be considered for such neoplasms, when their histopathologic and EUS features are favorable.

This study has several limitations, inherent to its retrospective and multicentric nature, with a very long recruitment period (more than 30 years), implying non-uniform and non-standardized therapeutic approaches to patients, in addition to the absence of disease-free survival data. However, it is a relatively large series for a very rare neoplastic disease, with a histologic review of all cases with updated WHO 2019 criteria.

In conclusion, Amp-NETs and Amp-NECs show different clinico-pathologic features and divergent prognosis, indicating two distinct neoplastic entities. In Amp-NETs, patient age > 60 years, WHO tumor grade G2, small vessel invasion, pT3 stage, having > 3 LNMs, and distant metastasis are determinants of adverse prognosis, while tumor site and size are predictors of LNMs, in addition to small vessel invasion. These factors should be considered when personalized treatment strategies are discussed.

## Data Availability

All data generated or analyzed during this study are included in this article. Further enquiries can be directed to the corresponding author.

## References

[1] Delle Fave G, Kwekkeboom DJ, Van Cutsem E, Rindi G, Kos-Kudla B, Knigge U, Sasano H, Tomassetti P, Salazar R, Ruszniewski P; Barcelona Consensus Conference participants. ENETS Consensus Guidelines for the management of patients with gastroduodenal neoplasms (2012) Neuroendocrinology 95:74–87. 10.1159/000335595 10.1159/000335595 22262004

[2] Hartel M, Wente MN, Sido B, Friess H, Büchler MW (2005). Carcinoid of the ampulla of Vater. J Gastroenterol Hepatol.

[3] Zhang XF, Wu XN, Tsilimigras DI, Poultsides G, Rocha F, Abbott DE, Fields R, Idrees K, Cho C, Maithel SK, Pawlik TM, other members of the US Neuroendocrine Tumor Study Group (2019). Duodenal neuroendocrine tumors: Impact of tumor size and total number of lymph nodes examined. J Surg Oncol.

[4] Schmocker RK, Wright MJ, Ding D, Javed AA, Cameron JL, Lafaro K, Burns WR, He J, Wolfgang CL, Burkhart RA (2021). Duodenal, ampullary, and pancreatic neuroendocrine tumors: Oncologic outcomes are driven by tumor biology and tissue of origin. J Surg Oncol.

[5] Pedicone R, Adham M, Hervieu V, Lombard-Bohas C, Guibal A, Scoazec JY, Chayvialle JA, Partensky C (2009). Long-term survival after pancreaticoduodenectomy for endocrine tumors of the ampulla of Vater and minor papilla. Pancreas.

[6] Untch BR, Bonner KP, Roggin KK, Reidy-Lagunes D, Klimstra DS, Schattner MA, Fong Y, Allen PJ, D'Angelica MI, DeMatteo RP, Jarnagin WR, Kingham TP, Tang LH (2014) Pathologic grade and tumor size are associated with recurrence-free survival in patients with duodenal neuroendocrine tumors. J Gastrointest Surg 18:457–462; discussion 462–3. 10.1007/s11605-014-2456-x 10.1007/s11605-014-2456-x 24448999

[7] Margonis GA, Samaha M, Kim Y, Postlewait LM, Kunz P, Maithel S, Tran T, Berger N, Gamblin TC, Mullen MG, Bauer TW, Pawlik TM (2016). A Multi-institutional Analysis of Duodenal Neuroendocrine Tumors: Tumor Biology Rather than Extent of Resection Dictates Prognosis. J Gastrointest Surg.

[8] Dogeas E, Cameron JL, Wolfgang CL, Hirose K, Hruban RH, Makary MA, Pawlik TA, Choti MA (2017). Duodenal and Ampullary Carcinoid Tumors: Size Predicts Necessity for Lymphadenectomy. J Gastrointest Surg.

[9] Massironi S, Campana D, Partelli S, Panzuto F, Rossi RE, Faggiano A, Brighi N, Falconi M, Rinzivillo M, Delle Fave G, Colao AM, Conte D (2018). Heterogeneity of Duodenal Neuroendocrine Tumors: An Italian Multi-center Experience. Ann Surg Oncol.

[10] Gay-Chevallier S, de Mestier L, Perinel J, Forestier J, Hervieu V, Ruszniewski P, Millot I, Valette PJ, Pioche M, Lombard-Bohas C, Subtil F, Adham M, Walter T (2021). Management and Prognosis of Localized Duodenal Neuroendocrine Neoplasms. Neuroendocrinology.

[11] Clements WM, Martin SP, Stemmerman G, Lowy AM (2003). Ampullary carcinoid tumors: rationale for an aggressive surgical approach. J Gastrointest Surg.

[12] Randle RW, Ahmed S, Newman NA, Clark CJ (2014). Clinical outcomes for neuroendocrine tumors of the duodenum and ampulla of Vater: a population-based study. J Gastrointest Surg.

[13] Nießen A, Bergmann F, Hinz U, Schimmack S, Hackert T, Büchler MW, Strobel O (2020). Surgical resection for duodenal neuroendocrine neoplasia: Outcome, prognostic factors and risk of metastases. Eur J Surg Oncol.

[14] Exarchou K, Moore AR, Smart HL, Duckworth CA, Howes N, Pritchard DM (2021). A "Watch and Wait" Strategy Involving Regular Endoscopic Surveillance Is Safe for Many Patients with Small, Sporadic, Grade 1, Non-Ampullary, Non-Functioning Duodenal Neuroendocrine Tumours. Neuroendocrinology.

[15] Ruff SM, Standring O, Wu G, Levy A, Anantha S, Newman E, Karpeh MS, Nealon W, Deutsch GB, Weiss MJ, DePeralta DK (2021). Ampullary Neuroendocrine Tumors: Insight into a Rare Histology. Ann Surg Oncol.

[16] Vanoli A, La Rosa S, Klersy C, Grillo F, Albarello L, Inzani F, Maragliano R, Manca R, Luinetti O, Milione M, Doglioni C, Rindi G, Capella C, Solcia E (2017). Four Neuroendocrine Tumor Types and Neuroendocrine Carcinoma of the Duodenum: Analysis of 203 Cases. Neuroendocrinology.

[17] Vanoli A, Albarello L, Uncini S, Fassan M, Grillo F, Di Sabatino A, Martino M, Pasquali C, Milanetto AC, Falconi M, Partelli S, Doglioni C, Schiavo-Lena M, Brambilla T, Pietrabissa A, Sessa F, Capella C, Rindi G, La Rosa S, Solcia E, Paulli M (2019). Neuroendocrine Tumors (NETs) of the Minor Papilla/Ampulla: Analysis of 16 Cases Underlines Homology With Major Ampulla NETs and Differences From Extra-Ampullary Duodenal NETs. Am J Surg Pathol.

[18] Okubo Y, Yoshioka E, Suzuki M, Washimi K, Kawachi K, Kameda Y, Yokose T (2018). Diagnosis, Pathological Findings, and Clinical Management of Gangliocytic Paraganglioma: A Systematic Review. Front Oncol.

[19] Rindi G, Mete O, Uccella S, Basturk O, La Rosa S, Brosens LAA, Ezzat S, de Herder WW, Klimstra DS, Papotti M, Asa SL (2022). Overview of the 2022 WHO Classification of Neuroendocrine Neoplasms. Endocr Pathol.

[20] Panzuto F, Massironi S, Partelli S, Campana D, Rinzivillo M, Invernizzi P, Andreasi V, Lamberti G, Falconi M (2020). Gastro-entero-pancreatic neuroendocrine neoplasia: The rules for non-operative management. Surg Oncol.

[21] de Mestier L, Lepage C, Baudin E, Coriat R, Courbon F, Couvelard A, Do Cao C, Frampas E, Gaujoux S, Gincul R, Goudet P, Lombard-Bohas C, Poncet G, Smith D, Ruszniewski P, Lecomte T, Bouché O, Walter T, Cadiot G, Thésaurus National de Cancérologie Digestive (TNCD) (2020). Digestive Neuroendocrine Neoplasms (NEN): French Intergroup clinical practice guidelines for diagnosis, treatment and follow-up (SNFGE, GTE, RENATEN, TENPATH, FFCD, GERCOR, UNICANCER, SFCD, SFED, SFRO, SFR). Dig Liver Dis.

[22] Hwang S, Lee SG, Lee YJ, Han DJ, Kim SC, Kwon SH, Ryu JH, Park JI, Lee HJ, Choi GW, Yu ES (2008). Radical surgical resection for carcinoid tumors of the ampulla. J Gastrointest Surg.

[23] Milanetto AC, Pasquali C, Da Broi M, Brambilla T, Capretti G, Zerbi A (2018). Ampullary neuroendocrine neoplasms: surgical experience of a rare and challenging entity. Langenbecks Arch Surg.

[24] Garbrecht N, Anlauf M, Schmitt A, Henopp T, Sipos B, Raffel A, Eisenberger CF, Knoefel WT, Pavel M, Fottner C, Musholt TJ, Rinke A, Arnold R, Berndt U, Plöckinger U, Wiedenmann B, Moch H, Heitz PU, Komminoth P, Perren A, Klöppel G (2008). Somatostatin-producing neuroendocrine tumors of the duodenum and pancreas: incidence, types, biological behavior, association with inherited syndromes, and functional activity. Endocr Relat Cancer.

[25] WHO Classification of Tumours Editorial Board (2019). WHO Classification of Tumors: Digestive System Tumours.

[26] Elvebakken H, Perren A, Scoazec JY, Tang LH, Federspiel B, Klimstra DS, Vestermark LW, Ali AS, Zlobec I, Myklebust TÅ, Hjortland GO, Langer SW, Gronbaek H, Knigge U, Tiensuu Janson E, Sorbye H (2021). A Consensus-Developed Morphological Re-Evaluation of 196 High-Grade Gastroenteropancreatic Neuroendocrine Neoplasms and Its Clinical Correlations. Neuroendocrinology.

[27] Amin MB (2017). AJCC Cancer Staging Manual.

[28] Rossi RE, Milanetto AC, Andreasi V, Campana D, Coppa J, Nappo G, Rinzivillo M, Invernizzi P, Modica R, David A, Partelli S, Lamberti G, Mazzaferro V, Zerbi A, Panzuto F, Pasquali C, Falconi M, Massironi S, ItaNet (Italian Association for Neuroendocrine Tumours) study group (2021). Risk of preoperative understaging of duodenal neuroendocrine neoplasms: a plea for caution in the treatment strategy. J Endocrinol Invest.

[29] Albores-Saavedra J, Hart A, Chablé-Montero F, Henson DE (2010) Carcinoids and high-grade neuroendocrine carcinomas of the ampulla of vater: a comparative analysis of 139 cases from the surveillance, epidemiology, and end results program-a population based study Arch Pathol Lab Med 134:1692–6. 10.5858/2009-0697-OAR.1 10.5858/2009-0697-OAR.1 21043824

[30] Nassar H, Albores-Saavedra J, Klimstra DS (2005). High-grade neuroendocrine carcinoma of the ampulla of vater: a clinicopathologic and immunohistochemical analysis of 14 cases. Am J Surg Pathol.

[31] Rindi G, Klersy C, Albarello L, Baudin E, Bianchi A, Buchler MW, Caplin M, Couvelard A, Cros J, de Herder WW, Delle Fave G, Doglioni C, Federspiel B, Fischer L, Fusai G, Gavazzi F, Hansen CP, Inzani F, Jann H, Komminoth P, Knigge UP, Landoni L, La Rosa S, Lawlor RT, Luong TV, Marinoni I, Panzuto F, Pape UF, Partelli S, Perren A, Rinzivillo M, Rubini C, Ruszniewski P, Scarpa A, Schmitt A, Schinzari G, Scoazec JY, Sessa F, Solcia E, Spaggiari P, Toumpanakis C, Vanoli A, Wiedenmann B, Zamboni G, Zandee WT, Zerbi A, Falconi M (2018). Competitive Testing of the WHO 2010 versus the WHO 2017 Grading of Pancreatic Neuroendocrine Neoplasms: Data from a Large International Cohort Study. Neuroendocrinology.

[32] Vanoli A, La Rosa S, Miceli E, Klersy C, Maragliano R, Capuano F, Persichella A, Martino M, Inzani F, Luinetti O, Di Sabatino A, Sessa F, Paulli M, Corazza GR, Rindi G, Bordi C, Capella C, Solcia E (2018). Prognostic Evaluations Tailored to Specific Gastric Neuroendocrine Neoplasms: Analysis Of 200 Cases with Extended Follow-Up. Neuroendocrinology.

[33] Kasajima A, Konukiewitz B, Schlitter AM, Weichert W, Klöppel G (2022). An analysis of 130 neuroendocrine tumors G3 regarding prevalence, origin, metastasis, and diagnostic features. Virchows Arch.

[34] Klöppel G, La Rosa S (2018). Ki67 labeling index: assessment and prognostic role in gastroenteropancreatic neuroendocrine neoplasms. Virchows Arch.

[35] Burgart LJ, Chopp WV, Jain D (2021) College of American Pathologists Protocol for the Examination of Specimens From Patients with Well-Differentiated Neuroendocrine Tumors (Carcinoid Tumors) of the Duodenum and Ampulla of Vater.https://documents.cap.org/protocols/DuodAmp.NET_1.1.0.0.REL_CAPCP.pdf

[36] Dumitrascu T, Dima S, Herlea V, Tomulescu V, Ionescu M, Popescu I (2012). Neuroendocrine tumours of the ampulla of Vater: clinico-pathological features, surgical approach and assessment of prognosis. Langenbecks Arch Surg.

[37] Yang K, Yun SP, Kim S, Shin N, Park DY, Seo HI (2017). Clinicopathological features and surgical outcomes of neuroendocrine tumors of ampulla of Vater. BMC Gastroenterol.

[38] Iwasaki T, Nara S, Kishi Y, Esaki M, Shimada K, Hiraoka N (2017). Surgical treatment of neuroendocrine tumors in the second portion of the duodenum: a single center experience and systematic review of the literature. Langenbecks Arch Surg.

[39] Partelli S, Javed AA, Andreasi V, He J, Muffatti F, Weiss MJ, Sessa F, La Rosa S, Doglioni C, Zamboni G, Wolfgang CL, Falconi M (2018). The number of positive nodes accurately predicts recurrence after pancreaticoduodenectomy for nonfunctioning neuroendocrine neoplasms. Eur J Surg Oncol.

[40] Zaidi MY, Lopez-Aguiar AG, Dillhoff M, Beal E, Poultsides G, Makris E, Rocha F, Crown A, Idrees K, Marincola Smith P, Nathan H, Beems M, Abbott D, Barrett JR, Fields RC, Davidson J, Cardona K, Maithel SK (2019). Prognostic Role of Lymph Node Positivity and Number of Lymph Nodes Needed for Accurately Staging Small-Bowel Neuroendocrine Tumors. JAMA Surg.

[41] Zhang XF, Xue F, Dong DH, Lopez-Aguiar AG, Poultsides G, Makris E, Rocha F, Kanji Z, Weber S, Fisher A, Fields R, Krasnick BA, Idrees K, Smith PM, Cho C, Beems M, Lv Y, Maithel SK, Pawlik TM (2021). New Nodal Staging for Primary Pancreatic Neuroendocrine Tumors: A Multi-institutional and National Data Analysis. Ann Surg.

[42] Makhlouf HR, Burke AP, Sobin LH (1999). Carcinoid tumors of the ampulla of Vater: a comparison with duodenal carcinoid tumors. Cancer.

[43] Zyromski NJ, Kendrick ML, Nagorney DM, Grant CS, Donohue JH, Farnell MB, Thompson GB, Farley DR, Sarr MG (2001). Duodenal carcinoid tumors: how aggressive should we be?. J Gastrointest Surg.

[44] Witzigmann H, Loracher C, Geissler F, Wagner T, Tannapfel A, Uhlmann D, Caca K, Hauss J, Hehl JA (2002). Neuroendocrine tumours of the duodenum. Clinical aspects, pathomorphology and therapy. Langenbecks Arch Surg.

[45] Pavel M, Öberg K, Falconi M, Krenning EP, Sundin A, Perren A, Berruti A, Guidelines Committee ESMO (2020). Gastroenteropancreatic neuroendocrine neoplasms: ESMO Clinical Practice Guidelines for diagnosis, treatment and follow-up. Ann Oncol.

[46] Delle Fave G, O'Toole D, Sundin A, Taal B, Ferolla P, Ramage JK, Ferone D, Ito T, Weber W, Zheng-Pei Z, De Herder WW, Pascher A, Ruszniewski P, Vienna Consensus Conference Participants (2016). ENETS Consensus Guidelines Update for Gastroduodenal Neuroendocrine Neoplasms. Neuroendocrinology.

